# Supplementary material for: Resolving the full spectrum of human genome variation using Linked-Reads
Source: Genome Res. 2019 Apr;29(4):635–45. doi: 10.1101/gr.234443.118 (PMC6442396; doi:10.1101/gr.234443.118)
Supplement: Supplemental Material [file supp_gr.234443.118_Supplemental_longranger-2.2.zip › longranger-2.2.tar.bz2!/lr2.2-public/lib/go/src/code.google.com/vitess/docs/resources/presentations.html]

Vitess / Presentations and Videos


Toggle navigation

- Overview
- Guides
- Reference
- Blog
- GitHub

- #### Overview

  - What is Vitess
  - Scaling MySQL with Vitess
  - Key Concepts
- #### Getting Started

  - Run Vitess on Kubernetes
    - Custom Docker Build
  - Run Vitess Locally
- #### User Guide

  - Introduction- Client Libraries- Backing Up Data- Reparenting
        - Schema Management
        - VSchema Guide
        - Schema Swap (Tutorial)
        - Sharding
          - Horizontal Sharding (Tutorial, automated)
          - Horizontal Sharding (Tutorial, manual)
          - Sharding in Kubernetes (Tutorial, automated)
          - Sharding in Kubernetes (Tutorial, manual)
        - Vitess Sequences
        - MySQL Server Protocol
        - Vitess and Replication
        - Update Stream
        - Row Based Replication
        - Topology Service
        - Transport Security Model
        - Launching
          - Scalability Philosophy
          - Production Planning
          - Server Configuration
          - 2PC Guide
          - Troubleshooting
        - Upgrading
- #### Reference Guides

  - Vitess API- vtctl Commands
- #### Other Resources

  - Presentations- Blog- Roadmap
- #### Contributing

  - Contributing to Vitess- GitHub Workflow- Code Reviews
- GitHub

# Presentations and Videos

- #### Overview

  - What is Vitess
  - Scaling MySQL with Vitess
  - Key Concepts
- #### Getting Started

  - Run Vitess on Kubernetes
    - Custom Docker Build
  - Run Vitess Locally
- #### User Guide

  - Introduction- Client Libraries- Backing Up Data- Reparenting
        - Schema Management
        - VSchema Guide
        - Schema Swap (Tutorial)
        - Sharding
          - Horizontal Sharding (Tutorial, automated)
          - Horizontal Sharding (Tutorial, manual)
          - Sharding in Kubernetes (Tutorial, automated)
          - Sharding in Kubernetes (Tutorial, manual)
        - Vitess Sequences
        - MySQL Server Protocol
        - Vitess and Replication
        - Update Stream
        - Row Based Replication
        - Topology Service
        - Transport Security Model
        - Launching
          - Scalability Philosophy
          - Production Planning
          - Server Configuration
          - 2PC Guide
          - Troubleshooting
        - Upgrading
- #### Reference Guides

  - Vitess API- vtctl Commands
- #### Other Resources

  - Presentations- Blog- Roadmap
- #### Contributing

  - Contributing to Vitess- GitHub Workflow- Code Reviews

## Percona Live 2016

Sugu and Anthony
showed what it looks like to use Vitess now that
Keyspace IDs can be
completely hidden from the application. They gave a live demo of
resharding the Guestbook sample app,
which now knows nothing about shards, and explained how new features in VTGate
make all of this possible.

download slides

## CoreOS Meetup, January 2016

Vitess team member Anthony Yeh's talk at
the January 2016 CoreOS Meetup
discussed challenges and techniques for running distributed databases
within Kubernetes, followed by a deep dive into the design trade-offs
of the Vitess on Kubernetes
deployment templates.

download slides

## Oracle OpenWorld 2015

Vitess team member Anthony Yeh's talk at
Oracle OpenWorld 2015 focused on
what the Cloud Native Computing paradigm means when
applied to MySQL in the cloud. The talk also included a deep dive into
transparent, live resharding, one of the key
features of Vitess that makes it well-adapted for a Cloud Native environment.

download slides

## Percona Live 2015

Vitess team member Anthony Yeh's talk at
Percona Live 2015 provided an overview of Vitess as well as an explanation
of how Vitess has evolved to live in a containerized world with
Kubernetes and Docker.

download slides

## Google I/O 2014 - Scaling with Go: YouTube's Vitess

In this talk, Sugu Sougoumarane
from the Vitess team talks about how Vitess
solved YouTube's scalability problems as well as about tips and techniques
used to scale with Go.

Contact: vitess@googlegroups.com  **·**  
Announcements  **·**  
© 2017 Vitess powered by Google Inc
